# Surgical modeling of Chiari-like malformation in rats: Insights from canine morphology

**DOI:** 10.1371/journal.pone.0310505

**Published:** 2024-09-19

**Authors:** Jae-Hwan Jung, Chang-Hyeon Cho, Sang-Yoon Shin, Eun-Ji Chung, Da-Eun Seo, Woo-Suk Kim, Hun-Young Yoon

**Affiliations:** 1 Department of Veterinary Surgery, College of Veterinary Medicine, Konkuk University, Seoul, Republic of Korea; 2 Department of Veterinary Radiology, College of Veterinary Medicine, Konkuk University, Seoul, Republic of Korea; 3 Department of Anatomy, College of Veterinary Medicine, and Veterinary Science Research Institute, Konkuk University, Seoul, Republic of Korea; 4 KU Center for Animal Blood Medical Science, Konkuk University, Seoul, Republic of Korea; Wrocław University of Environmental and Life Sciences: Uniwersytet Przyrodniczy we Wroclawiu, POLAND

## Abstract

**Background:**

Chiari-like malformation in dogs and Chiari malformation type 1 in humans are conditions characterized by a relatively small caudal cranial fossa, leading to cerebellar herniation. This study aimed to develop a rat model of Chiari-like malformation using surgical techniques based on morphological characteristics observed in dogs.

**Methods:**

Endocranial magnetic resonance images of both normal dogs and dogs diagnosed with Chiari-like malformation were retrospectively analyzed. Measurements of the caudal cranial fossa volume, rostral and medial fossa volume, and volume index were taken. The differences in caudal cranial fossa volume and volume index between normal dogs and those diagnosed with Chiari-like malformation were then utilized to create a rat model of Chiari-like malformation through surgical intervention. The measurements were conducted on both the rat Chiari-like malformation models and normal rats, with each measurement taken twice and the mean values calculated.

**Results:**

Significant differences were found between normal dogs and dogs diagnosed with Chiari-like malformation in terms of the volume of the caudal cranial fossa (27.62% reduction) and the volume index (23.36% reduction) (p<0.05). These differences were used to develop a rat model, which also showed significant reductions in both caudal cranial fossa volume (29.52%) and volume index (28.30%) compared to normal rats (p<0.05). The condition in the rat model was confirmed through magnetic resonance imaging, which revealed cerebellar herniation into the foramen magnum.

**Conclusions:**

The study successfully established a rat model of Chiari-like malformation that accurately reproduces the morphological features observed in dogs. This model potentially serves as a valuable tool for investigating the pathological mechanisms and potential therapeutic approaches for Chiari-like malformation in veterinary medicine.

## Introduction

In human medicine, Chiari malformations encompass a diverse group of malformations that affect the posterior fossa and craniovertebral junction. These malformations are characterized by the herniation of the cerebellar tonsils below the foramen magnum, occasionally accompanied by varying degrees of descent into the brainstem [1]. Analogous to Chiari type 1, the most prevalent type of Chiari malformation discovered in small dogs was named Chiari-like malformation (CLM) by the International Veterinary Working Group [1]. CLM involves a relatively small caudal cranial fossa (CCF) due to morphological malformations of the skull and craniocervical junction, leading to a discrepancy between the CCF volume and brain parenchyma [2, 3]. This discrepancy results in CCF overcrowding, causing the caudal displacement of the cerebellum through the foramen magnum [2–4]. Cerebellar herniation due to skull insufficiency has been associated with secondary syringomyelia (SM) by altering normal cerebrospinal fluid (CSF) flow, which is caused by the obstruction of the dorsal craniocervical subarachnoid space and medullary kinking [5, 6].

Magnetic resonance imaging (MRI), which assesses linear and volumetric brain and cervical changes through T1-weighted (T1W) and T2-weighted (T2W) sagittal and transverse imaging [2, 6], serves as the gold standard for CLM diagnosis. Sagittal T2W images are the most valuable sequences [5].

The diagnosis is made by identifying abnormal findings, such as caudal cerebellar herniation, caudal cerebellar compression due to occipital dysplasia, and CSF attenuation [2, 5].

Chiari malformation poses an important challenge in human and veterinary research due to the lack of clear pathological mechanisms and treatment methods. Moreover, methods for inducing CLM through acquired means in experimental animals are lacking. In particular, to the best of our knowledge, there is no known method for inducing CLM. The current situation is worsened by a lack of appropriate animal models for experimental research, hindering an in-depth understanding of the pathological mechanisms of Chiari malformation and impeding the development of effective treatment strategies. Therefore, this study aims to address this knowledge gap by presenting the development of a rat CLM model, which can serve as a fundamental tool for advancing future research on Chiari disease. Specifically, by using a surgical method, this study proposes a reliable and novel approach for creating the first CLM model.

## Materials and methods

### Presurgical CCF volume measurement in dogs

In the case of canine CLM, MRI was used to determine the extent of reduction in CCF volume compared with that in normal dogs. Since body weight and sex influence absolute volume, resulting in bias, the relative volume of the CCF for the rostral and medial fossa (RMF) was calculated using the volume index (VI) and compared with that in normal dogs [7].

#### Case selection

Dogs were retrospectively selected from electronic patient records at the Konkuk University Veterinary Medical Teaching Hospital from 2020 to 2023. The selection criteria were established by reviewing MRIs (Signa Hdxt, 1.5 T MRI, GE HealthCare, Chicago, IL, USA), including 2D MRI sequences (T2W transverse, T2W sagittal; slice thickness 2 mm, interslice gap 0 mm) and 3D MRI sequences (T2W CUBE transverse, T2W CUBE sagittal, T1W FSGPR transverse; slice thickness 1 mm, interslice gap 0 mm).

The eligibility criteria were as follows and were the same for all breeds: exclusion of immature dogs (<1 year old) due to potential incomplete skull growth and exclusion of dogs weighing <2 or >8 kg to ensure weight-matched groups. Dogs with parenchymal space-occupying lesions or other conditions that elevate intracranial pressure were also excluded [8]. Transverse scans were required to include the cribriform plate rostrally and the first cervical spinal cord segment caudally, whereas midsagittal scans had to encompass the whole brain parenchyma and the initial three cervical spine vertebrae [8]. CLM was defined by the presence of caudal cerebellar herniation into the foramen magnum or indentation by the supraoccipital bone, irrespective of the presence of SM [8].

**Fig 1:**
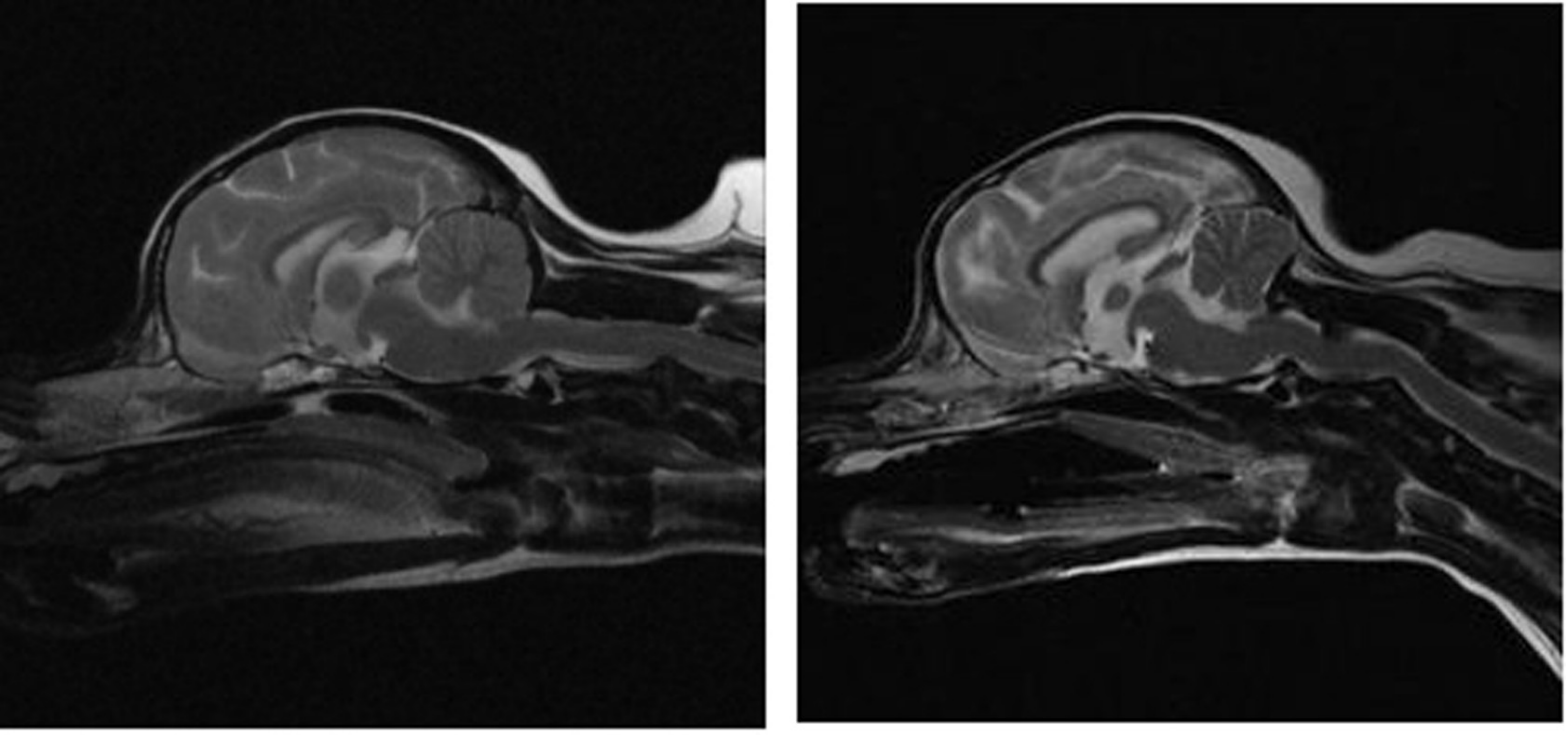
T2-weighted sagittal view magnetic resonance (MR) images of dog brains. (A) Dogs with normal brains. (B) Dogs diagnosed with Chiari-like malformation.

Eight normal dogs (dog-control [DC] group) and eight dogs diagnosed with CLM (dog-Chiari [DH] group) that satisfied the above criteria were included in the study (Fig 1). Guardians were informed separately that the imaging data would be used for research and experimental purposes, and their written informed consent was obtained. The retrospective analysis of the archived images was approved by the Institutional Animal Care and Use Committee of Konkuk University, Seoul, Republic of Korea (approval number: KU23177).

#### Volume measurement through MRI

The MRI reports of each dog were reviewed, and CCF and RMF volumes were measured by the same board-certified radiologist. To minimize measurement bias, each subject was assigned a study number, and information regarding breed, age, sex, and weight was provided to the measurer in a blinded manner for volumetric measurements.

Images were processed for volume rendering using image analysis and scientific visualization software (3D Slicer 5.4.0, 2023.8.19), which provides image segmentation and 3D volume rendering functions. Manual image masking was performed to enhance the accuracy of segmentation [9, 10].

**Fig 2:**
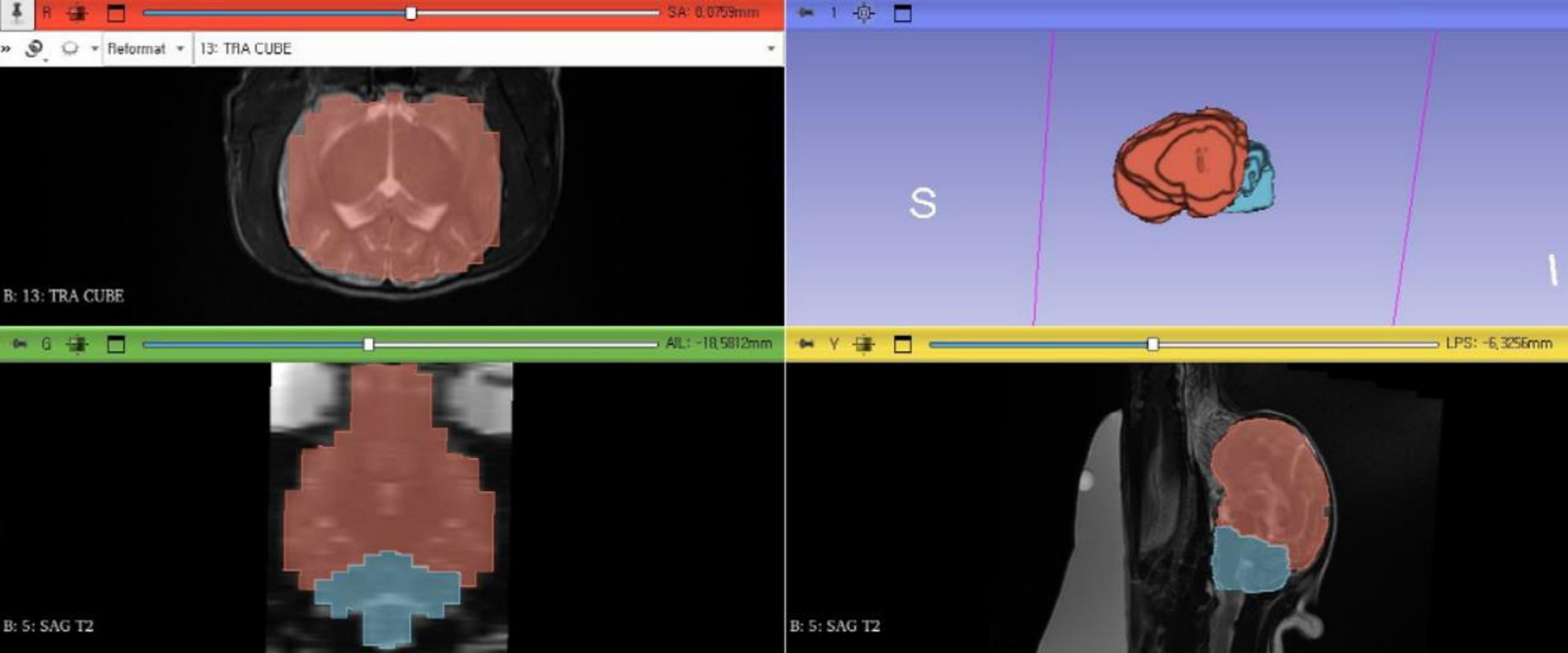
Screenshot of 3D SLICER 5.4.0. Image processing for volume rendering of the rostral and medial fossa (red region) and caudal cranial fossa (blue region) of an 8-year-old spayed female Pomeranian.

The image-processing method is shown in Fig 2. First, the MRI underwent segmentation slice by slice, with voxels corresponding to the anatomical structures of the CCF and RMF in each image identified. During this process, all brain volumes were designated to include the ventricular system volume [11]. The cranial boundary of the CCF volume was set along the contour of the rostral aspect of the cerebellum and the line connecting the point of contact between the cerebellum and brainstem to the rostral border of the pons [11]. The caudal boundary was defined as the line extending from the intercondylar incisures to the most caudal point of the foramen magnum [11]. CCF volume was calculated by combining the cerebellar and brainstem parenchyma volumes with the subarachnoid space volume [11]. The RMF volume was determined by adding the cerebral volume and subarachnoid space volume surrounding the cerebrum [11].

Subsequently, the segmented voxel values were masked to clarify the boundaries of anatomical structures [7]. The resulting mask contained information on all selected anatomical structures, and a 3D model was generated by performing volume rendering in combination with the original data and polygonal surface reconstruction algorithms [7, 9]. Volume measurements were performed using computer software to minimize calculation errors. The absolute volumes of the CCF and RMF in normal dogs and those in dogs with CLM were measured twice, and the average values were calculated. To minimize bias related to sex and body weight, which may influence absolute volume, the VI, which is the volume of the caudal fossa relative to that of the RMF, was additionally calculated for normalization. VI values were calculated twice, and the average value was obtained for each measurement.

### Creation of a rat CLM model through surgical methods

#### Animals

Sixteen male Sprague‒Dawley (SD) rats weighing 210–230 g and aged 7 weeks were purchased from DBL Co., Ltd., (Chungbuk, South Korea). The rats were acclimatized to the environment for 1 week. Throughout the experiment, they were individually housed in ventilated cages at 22 ± 2°C with 55 ± 5% relative humidity and subjected to a 12-h light/12-h dark cycle. The rats had *ad libitum* access to water and food. This study was approved by the Institutional Animal Care and Use Committee of Konkuk University (approval number: KU23177).

#### Experimental group

The rats were divided into two groups: a surgically induced CLM rat model group (rat-Chiari [RH] group, n = 8) and a healthy control group (rat-control [RC] group, n = 8). All surgical procedures were performed under aseptic conditions. By comparing the RH and RC groups, this study aimed to determine whether the CCF volume reduction in the RH group exceeded that in the DH group, as reported in previous studies. MRI was performed 2 weeks after rat CLM surgery.

#### Rat CLM model creation surgery

**Fig 3:**
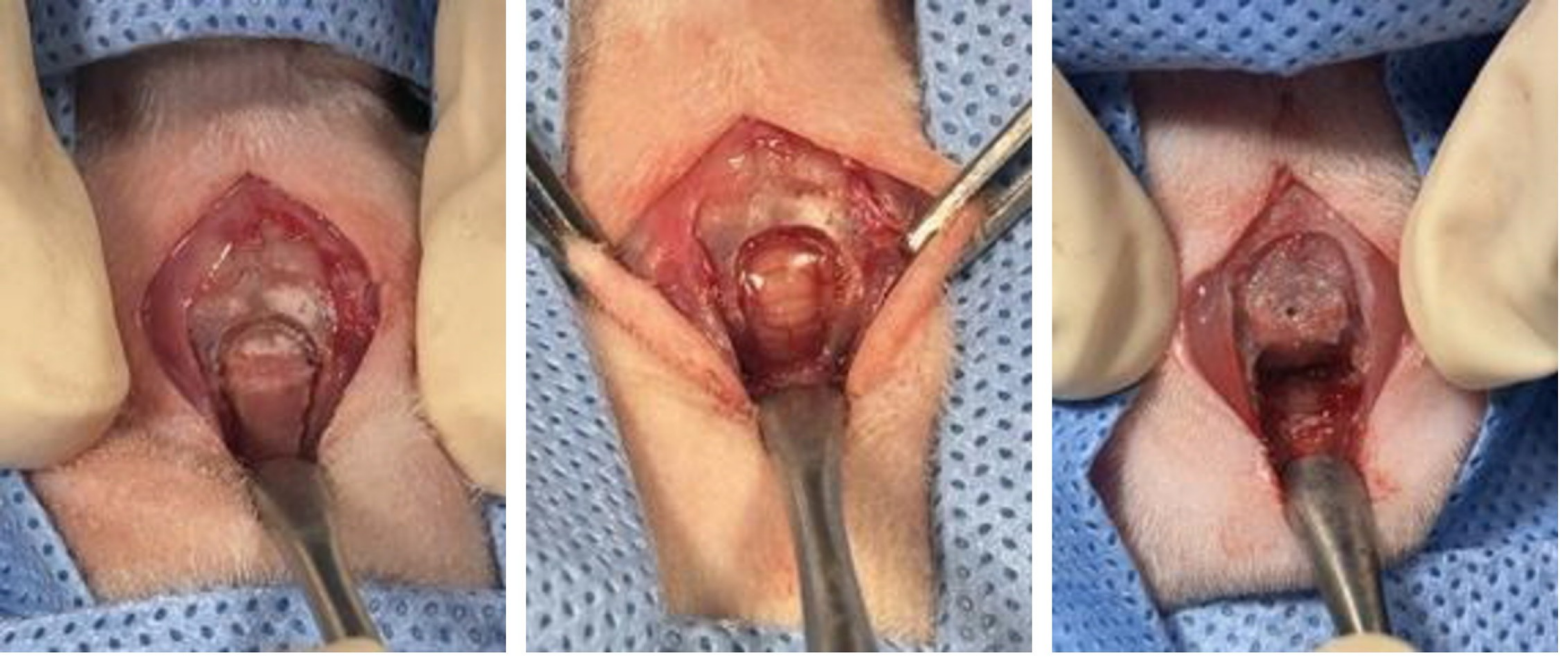
Surgical procedure for creating the Chiari-like malformation (CLM) model in 8-week-old male rats. (A, B) Using a carbide bur (FG 1/2, MANI Inc., Japan), partial resection of the occipital and interparietal bones was performed vertically from the diametric endpoints of the foramen magnum, 3 mm rostral to the external occipital protuberance, and 2 mm caudal to the lambda. (C) Subsequently, a small hole with a diameter of 0.6 mm was drilled, and the protruding part (approximately 3 mm) of the resected occipital bone fragment was positioned to overlap with the parietal and interparietal bones, which comprise the skull.

Male SD rats aged 8 weeks and weighing 240–270 g were used to establish the rat CLM model. The animals were placed in a small induction chamber and anesthetized with 4% isoflurane in oxygen for 2 min [12]. After induction, the rats were transferred to the surgical table and maintained with 1–2% isoflurane via a nose hose [12]. The surgical area was shaved and sterilized using povidone and alcohol. A skin incision was made approximately 2 cm from the bregma to the atlas using a No. 15 blade. Once it was confirmed that the periosteum covered the calvarium, a sagittal midline periosteal incision was made, and the periosteum was laterally elevated using a periosteal elevator. By separating the muscles attached to the occipital bone, the foramen magnum was exposed along with the interparietal and occipital bones. Using a high-speed dental handpiece equipped with a 0.6-mm-diameter carbide bur (FG 1/2, MANI Inc., Tochigi, Japan), the occipital bone and part of the interparietal bone were removed perpendicularly from both ends of the diameter of the foramen magnum, 3 mm rostral from the external occipital protuberance and 2 mm caudal from the lambda (Fig 3A and 3B).

At this stage, the burring rate did not exceed 1,000 rpm, and constant irrigation with sterile normal saline was maintained to prevent thermal damage [12]. Following removal, the bone fragments were placed in an NaCl (0.9%) solution, and the brain surface was regularly hydrated with NaCl solution. A small hole (0.6 mm diameter) was drilled using a high-speed dental handpiece equipped with a 0.6-mm-diameter carbide bur (FG 1/2, MANI Inc.) positioned 1 mm from the anterior border of the resected occipital bone fragment. The 3 mm protruding part of the resected occipital bone fragment was then overlapped with the parietal and interparietal bones that make up the skull and secured using Vetbond™ (3M Company, Saint Paul, MN, USA) (Fig 3C). The periosteum was closed using simple interrupted 5–0 absorbable monofilament sutures (Monocryl®, Ethicon, Raritan, NJ, USA). Skin closure was performed using 4–0 nonabsorbable monofilament sutures (Dafilon®, Melsungen, Germany).

Postoperatively, cefotaxime (30 mg/kg, SC, once daily), enrofloxacin (5 mg/kg, SC, once daily), tramadol (10 mg/kg, SC, once daily), and meloxicam (2 mg/kg, SC, once daily) were administered for a period of 2 weeks. The surgical site was disinfected daily with a 10% povidone-iodine solution for the same duration.

#### CLM diagnosis and volume measurements using MRI

**Fig 4:**
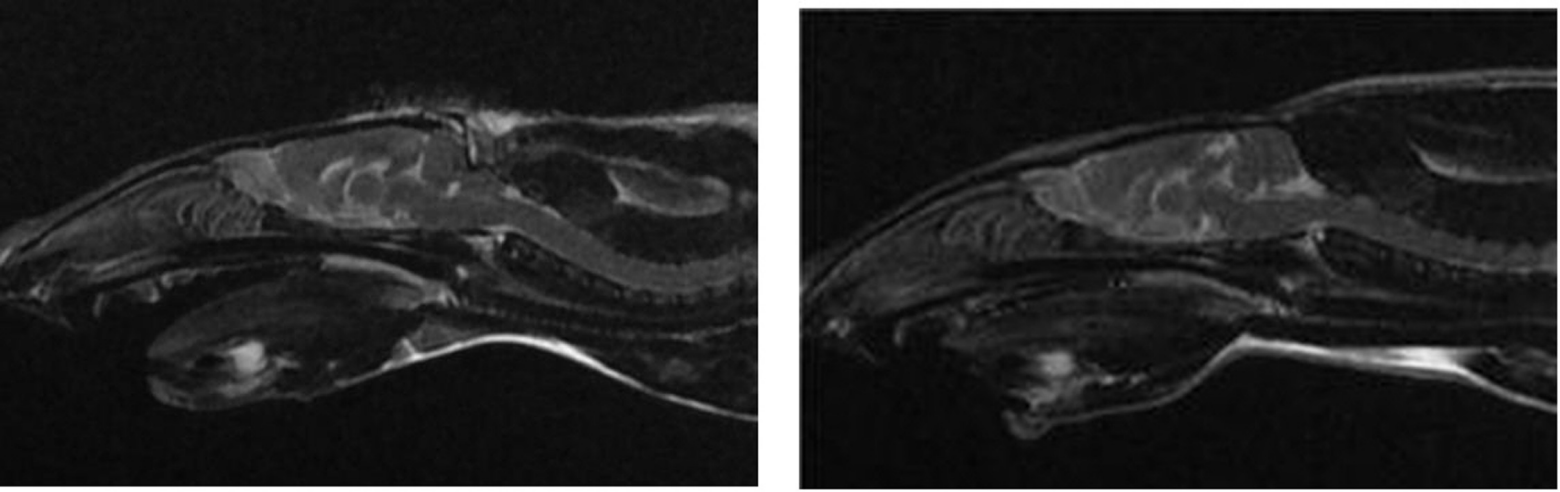
T2-weighted sagittal magnetic resonance image of the brain of a 10-week-old male rat. (A) Rat Chiari-like malformation (CLM) model in which cerebellar herniation into the foramen magnum is induced by surgically reducing the caudal cranial fossa volume. (B) Rat with a normal brain.

Two weeks after surgery, eight 10-week-old rats with CLM underwent MRI to assess CLM formation through cerebellar herniation. The RMF volume, CCF volume, and VI were measured simultaneously (Fig 4A). Subsequently, eight 10-week-old rats in the RC group underwent MRI to measure RMF volume, CCF volume, and VI (Fig 4B). All images were acquired using a 1.5 T MRI scanner (Signa Hdxt) with a knee coil (8 channels). The MRI sequences included the 2D T2W sagittal plane, 3D T2W CUBE transverse plane, 3D T2W CUBE sagittal plane, and 3D T1W FSPGR transverse plane, all with a slice thickness of 1 mm and interslice gap of 0 mm. The imaging protocol and volume measurements were performed in the same manner as before. Each measurement was performed twice, and the mean values were calculated.

The formation of the CLM model was assessed based on the Chiari malformation/syringomyelia grading guidelines provided by the British Veterinary Association (BVA) to classify the severity of structural abnormalities [6] (Table 1).

**Table 1:** British Veterinary Association (BVA)/Kennel Club (KC) Chiari malformation/syringomyelia scheme.

Grade	Chiari-like malformation (CLM)
**Grade 0**	No Chiari malformation
**Grade 1**	Cerebellum indented (not rounded)
**Grade 2**	Cerebellum impacted into, or herniated through, the foramen magnum

Therefore, based on these criteria, if abnormal findings such as caudal cerebellar compression, cerebellar vermis herniation, and CSF attenuation due to occipital dysplasia were confirmed through appropriate MRI evaluation, a diagnosis of CLM was made [2, 5].

The CCF volume, RMF volume, and VI were measured in the DC and DH groups as described above. These measurements were conducted twice for both the RC and RH groups, and the average values were calculated.

#### Methods of anesthesia and analgesia

All surgical procedures were performed under general anesthesia to minimize pain and discomfort in the animals. Induction was achieved using 4% isoflurane in oxygen for 2 minutes, and maintenance anesthesia was provided through a nose hose using 1–2% isoflurane [12]. Postoperatively, analgesia was administered as follows: tramadol (10 mg/kg, SC, once daily) and meloxicam (2 mg/kg, SC, once daily) for 2 weeks to manage pain and inflammation. Enrofloxacin (5 mg/kg, SC, once daily) and cefotaxime (30 mg/kg, SC, once daily) were administered to prevent infection.

#### Methods of sacrifice

At the conclusion of the study, the rats were humanely euthanized. The euthanasia process adhered to the AVMA Guidelines for the Euthanasia of Animals [13]. The rats were first anesthetized with isoflurane inhalation until they were unresponsive to painful stimuli. This was then followed by the intracardiac administration of potassium chloride to ensure a swift and humane death [13].

#### Efforts to mitigate animal suffering

Throughout the study, efforts were made to minimize any potential suffering of the animals. This involved regularly monitoring the rats for signs of pain, distress, or abnormal behavior, and taking necessary interventions to address these issues. Postoperative care included daily administration of analgesics, monitoring for signs of infection or complications at the surgical site, and daily disinfection of the surgical area using a 10% povidone-iodine solution. All procedures and handling of the animals were performed by trained personnel under the supervision of a veterinarian to ensure the highest standards of animal welfare.

#### Compliance with the Animal Research: Reporting of In Vivo Experiments (ARRIVE) guidelines

This study was conducted in compliance with the ARRIVE guidelines to ensure the highest standards of ethical treatment and reporting in animal research [14]. The guidelines were followed to enhance the reproducibility and transparency of the experimental methods, including detailed descriptions of the animal model, experimental procedures, and efforts to minimize animal suffering [14].

### Statistical analysis

All statistical analyses were conducted using the SPSS (ver. 26.0) commercial software package. The normality of the distribution was assessed using the Shapiro‒Wilk test, and descriptive statistics, including the mean ± standard deviation, as well as quartiles (Q1, Q2, Q3), are presented. Homogeneity between the experimental and control groups was assessed using the Chi-squared test and Mann‒Whitney U test. Differences in RMF, CCF, and VI (CCF/RMF) between the experimental and control groups were examined using the Mann–Whitney U test. The similarity in the decrease in CCF and VI (CCF/RMF) ratios between the dog and rat groups was evaluated using permutation tests in addition to percentages. The significance level was set at p<0.05.

## Results

### General characteristics of the DC and DH groups

The study included Maltese dogs, with 4 (50.0%) in the DC group and 4 (50.0%) in the DH group, and Pomeranian dogs, with 2 (66.7%) in the DC group and 1 (33.3%) in the DH group. The sex distribution comprised four (50%) castrated males and four (50%) spayed females in the DC and DH groups. The mean age was 10.13 ± 3.64 years in the DC group and 6.88 ± 3.18 years in the DH group, while the mean weight was 3.81 ± 1.55 kg in the DC group and 3.23 ± 0.53 kg in the DH group. The general characteristics of the DC and DH groups appeared to be homogeneous across all parameters (Table 2).

**Table 2:** Comparison of general characteristics and homogeneity tests between the dog-control (DC) and dog-Chiari (DH) groups.

Classification		n (%)	DC group (n = 8)	DH group (n = 8)	*χ*^2^/Z (*p*)
			n (%)	n (%)	
	Bichon frise	1 (6.3)	0 (0.0)	1 (100.0)	
	Dachshund	1 (6.3)	1 (100.0)	0 (0.0)	
	Longhair chihuahua	1 (6.3)	0 (0.0)	1 (100.0)	5.116 (1.000)
**Breed**	Maltese	8 (49.6)	4 (50.0)	4 (50.0)	
	Pomeranian	3 (18.9)	2 (66.7)	1 (33.3)	
	Silky terrier	1 (6.3)	0 (0.0)	1 (100.0)	
	Yorkshire terrier	1 (6.3)	1 (100.0)	0 (0.0)	
**Sex**	Castrated male	8 (50.0)	4 (50.0)	4 (50.0)	0.000
	Spayed female	8 (50.0)	4 (50.0)	4 (50.0)	(1.000)
**Age**	(Years) Mean ± SD (Median, Q1, Q3)	16 (100.0)	10.13 ± 3.64 (10.5, 8, 12)	6.88 ± 3.18 (8, 3.5, 9)	−1.703 (0.105)
**Weight**	(kg) Mean ± SD (Median, Q1, Q3)	″	3.81 ± 1.55 (3.6, 2.5, 5.2)	3.23 ± 0.53 (3.1, 2.8, 3.7)	−0.316 (0.798)

• *χ*^2^: Chi-squared test (Fisher’s exact test), Z: Mann‒Whitney U test.

DC, dog-control; DH, dog-Chiari; SD, standard deviation.

### Differences in the RMF volume, CCF volume, and VI between the DC and DH groups

Table 3 displays the differences in the RMF volume, CCF volume, and VI (CCF/RMF) between the DC and DH groups (S1 Fig).

**Table 3:** Differences in the RMF volume, CCF volume, and VI between the DC and DH groups.

Classification		DC group (n = 8)	DH group (n = 8)	Z (*p*)
		Mean ± SD (Median, Q1, Q3)	Mean ± SD (Median, Q1, Q3)	
**Mean value (mm** ^**3**^ **)**	RMF volume (mm^3^)	49,328.58 ± 7,614.09 (50536.33, 45344.70, 53331.35)	47,053.66 ± 6,264.07 (45963.13, 41273.00, 51963.05)	−1.050 (0.328)
	CCF	7,864.39 ± 1,990.45 (7625.38, 6084.09, 9224.03)	5,692.12 ± 1,030.13 (6029.05, 4624.09, 6662.12)	−2.100 (0.038)
	VI	0.1584 ± 0.02 (0.1569, 0.1349, 0.1794)	0.1214 ± 0.02 (0.1149, 0.1039, 0.1388)	−2.310 (0.021)

• Z: Mann‒Whitney U test.

RMF, rostral and medial fossa; CCF, caudal cranial fossa; VI, volume index; DC, dog-control; DH, dog-Chiari.

According to the average of the first and second measurements, RMF volume did not differ significantly between the DC and DH groups. However, a significant difference in the CCF volume was observed between the DC and DH groups (p = 0.038). VI also differed significantly between the DC and DH groups (p = 0.021) (Table 3).

### Differences in the RMF volume, CCF volume, and VI between the RC and RH groups

Table 4 presents the differences in the RMF volume, CCF volume, and VI between the RC and RH groups (S2 Fig). No significant difference was observed in the RMF volume between the RC and RH groups. However, the CCF volume significantly differed between the RC and RH groups (p<0.001). VI also differed significantly between the RC and RH groups (p<0.001).

**Table 4:** Differences in the RMF volume, CCF volume, and VI between the RC and RH groups.

Classification		RC group (n = 8)	RH group (n = 8)	Z (*p*)
		Mean ± SD (Median, Q1, Q3)	Mean ± SD (Median, Q1, Q3)	
**Mean value**	RMF (mm^3^)	1,447.78 ± 56.86 (1445.70, 1391.36, 1501.49)	1,422.24 ± 31.40 (1424.59, 1395.23, 1448.59)	−0.840 (0.442)
	CCF (mm^3^)	502.53 ± 13.25 (504.28, 495.95, 513.36)	354.17 ± 26.37 (353.21, 335.22, 356.65)	−3.361 (<0.001)
	VI (CCF/RMF)	0.3474 ± 0.01 (0.3431, 0.3380, 0.3610)	0.2491 ± 0.02 (0.2430, 0.2365, 0.2541)	−3.361 (<0.001)

• Z: Mann‒Whitney U test.

RMF, rostral and medial fossa; CCF, caudal cranial fossa; VI, volume index; RC, rat-control; RH, rat-Chiari.

### Comparison of the RMF, CCF, and VI ratios between the control and experimental groups

The changes in the RMF volume, CCF volume, and VI in the Chiari groups relative to the control groups, calculated as percentages (experimental group/control group × 100), are shown in Table 5. The changes in the RMF, CCF, and VI ratios were similar between dogs and rats (Table 5). By conducting permutation tests in addition to percentage comparisons, it was confirmed that the CCF (S3 Fig) and VI (S4 Fig) ratios exhibited similar characteristics between dogs and rats.

**Table 5:** Ratios of the DH group to the DC group and the RH group to the RC group.

Classification	RMF (%)	CCF (%)	VI (%) (CCF/RMF)
**DH group/DC group**	95.39	72.38	76.64
**RH group/RC group**	98.24	70.48	71.70

RMF, rostral and medial fossa; CCF, caudal cranial fossa; VI, volume index; RC, rat-control; RH, rat-Chiari.

## Discussion

Chiari malformation is a complex neurological abnormality that is prevalent in humans and animals. It presents an important medical challenge due to the lack of understanding of the pathological mechanisms and effective treatment methods [1, 5]. In this study, we aimed to develop a new animal model of CLM using rats. Unlike previously reported methods, the rat CLM model was induced through surgical intervention. Using small toy dog breeds weighing 2–8 kg, we observed that dogs with CLM had a decreased CCF volume ratio (approximately 27.62%) and a decreased VI value ratio (approximately 23.36%) compared to those in the control group. Following the surgical intervention, we used MRI to confirm whether the new rat Chiari model produced in this study could reproduce the CLM characteristics observed in dogs. All eight rats in this study were confirmed to have cerebellar herniation due to a smaller CCF volume, corresponding to CLM Grade 2 based on the BVA criteria [6]. Thus, we were able to confirm the effectiveness and reproducibility of our rat CLM model.

In human embryology, the mesoderm emerges during the third week of embryonic development, inducing the transformation from the ectoderm to the neuroectoderm and giving rise to the neural tube by the fourth week [1]. The skull and vertebral column, which are components of the axial skeleton, originate from the paraxial and lateral plate mesoderm and neural crest [1]. Considering these embryological processes, in 1995, Briner et al. [15] induced spina bifida in rats using valproic acid, while in 2005 and 2010, Barreto et al. [16] and Corti et al. [17] induced spinal dysraphism by opening the dura mater, respectively. However, most of these studies are limited to deriving or creating experimental animal models of Chiari type 2, a congenital form of the condition. To date, no experimental animal model of Chiari type 1 has been successfully developed. Therefore, this study proposes a novel approach to create an experimental animal model to improve our understanding of Chiari malformation, which has remained elusive due to the absence of an experimental animal model.

Like Chiari malformation in humans, this condition also affects dogs and is referred to as CLM in veterinary medicine because it resembles Chiari type 1 malformation in humans [1]. CLM is characterized by overcrowding of the brain parenchyma due to a reduction in the volume of the caudal cranial fossa, resulting in caudal displacement of the cerebellum and occasionally the brainstem into the foramen magnum [6]. This condition results in cerebellar compression, herniation, medullary kinking, obstruction of the dorsal craniocervical subarachnoid space, ventriculomegaly/hydrocephalus, turbulent CSF flow, and SM [2].

In humans, Chiari type 1 is diagnosed when one or both cerebellar tonsils protrude more than 5 mm below the foramen magnum, as measured by the McRae Line, a line drawn from the base to the opisthion, through MRI [18]. In veterinary medicine, the diagnosis of CLM refers to the CLM grading guidelines of the BVA and is classified as Grade 0 (normal), Grade 1 (mild CLM), or Grade 2 (substantial CLM), depending on the severity of the structural abnormality [6].

An experimental animal model of CLM was developed by inducing cerebellar herniation in rats by reducing the CCF volume, which is the cause of Chiari type 1 and CLM in both human and veterinary medicine. On sagittal T2W MR images, the herniated cerebellum in rats was found to obstruct the cisterna magna, indicating damage to the interface between the intracranial and intraspinal compartments, disrupting bidirectional CSF flow and CSF pulse pressure equilibration [19]. This leads to distorted viscoelastic support for the cerebellum and spinal cord [19]. Moreover, similar to what has been reported in human medicine, it is considered to induce secondary pathophysiological effects that cause damage and dysfunction of extradural ligaments, including myodural bridges, intradural dentate ligaments, and craniospinal suspension structures such as the arachnoid framework [19].

Recent claims suggest that Chiari type 1, the most prevalent form, should be reclassified as an acquired and potentially reversible abnormality from its previous classification as a congenital malformation [1, 20, 21]. There is evidence that Chiari type 1 is not congenital but rather a deformity or acquired abnormality [20]. First, Chiari type 1 is rarely found in newborns, and cases discovered in early childhood often exhibit a growth discrepancy between the cerebellum and the posterior cranial fossa after birth [20]. Histological examination of the cerebellar tonsils revealed neuronal disorganization or brainstem dysplasia in Chiari type 2, whereas in Chiari type 1, they typically appeared normal or exhibited minor necrosis due to pressure [20]. Finally, numerous studies have demonstrated that cerebellar tonsillar ectopia is reversibly corrected after the underlying cause (such as foramen magnum decompression) is treated, leading to morphological normalization of the hindbrain and cerebellar tonsils [20]. Therefore, the recent argument that Chiari type 1 should be called Chiari deformation rather than Chiari malformation in human medicine supports the validity of this study, which established a rat CLM model using an acquired method rather than an innate method.

There are various potential research areas in which the rat CLM model can be utilized. In the pursuit of unraveling the complexities of Chiari disease, the rat CLM model emerges as a pivotal asset, drawing on insights garnered from canine morphology. Through this model, a range of promising research paths come to light, each poised to advance our understanding and treatment strategies for this enigmatic condition. The rat CLM model offers a fertile ground for the development and evaluation of diverse treatment modalities aimed at combating Chiari disease. From pharmacological interventions to innovative surgical techniques and rehabilitative therapies, the model serves as a versatile testing ground for therapeutic innovation. Furthermore, the replication of key anatomical features observed in dogs within the rat CLM model provides a unique opportunity to dissect the intricate pathophysiological mechanisms underlying Chiari disease. By explaining the etiological and progressive pathways of the condition, researchers can pave the way for targeted interventions and personalized treatments. Additionally, the rat CLM model facilitates a thorough exploration of the neurological consequences associated with Chiari disease. By scrutinizing cerebellar abnormalities and their impact on neurological function, researchers can uncover the underlying biology of the disease, offering crucial insights into its clinical manifestations. Furthermore, the rat CLM model offers a robust platform for assessing the clinical translatability of novel therapeutic strategies. This crucial step connects preclinical findings with potential clinical applications, promoting the development of effective treatments for Chiari disease. In summary, the rat CLM model stands as a beacon of promise in the field of Chiari disease research, offering invaluable insights and avenues for therapeutic innovation. With its multifaceted usefulness, this model has the potential to revolutionize our understanding and management of Chiari-related pathologies.

In this rat CLM model, the resection and attachment of the occipital bone altered the position of the foramen magnum towards the cranial side, but the position of the atlas (C1 vertebra) remained the same. This alteration may limit the applicability of the model for studying morphological changes as the length of the craniocervical junction increases. However, it is important to note that in our model, herniation was indeed induced by altering the anatomy of the CCF rather than merely changing the position of the foramen magnum. The surgical intervention involved resecting and reattaching the occipital bone to decrease the CCF volume, causing cerebellar herniation into the foramen magnum, similar to what is seen in natural cases of CLM in dogs. To address the limitation of morphological changes and further validate the accuracy of our model, future comparative research is needed using methods to quantify CSF flow velocity and turbulence through cine MRI or phase-contrast MRI based on changes in the length of the craniocervical junction [2, 6]. Additionally, detailed comparisons of the degree of cerebellar crowding and other pathological features between our model and naturally occurring CLM cases will be crucial for confirming the relevance and applicability of the model. This study has several limitations that should be considered when interpreting the results. First, the rat CLM model developed in this study, although based on canine morphology, may not fully replicate the complexity of Chiari malformation type 1 in humans. The anatomical differences between rats and humans could limit the direct applicability of our findings to human conditions. Second, the sample size in this study was relatively small, which might limit the statistical power of our findings. Larger studies are needed to confirm our results. Third, this study primarily focused on short-term outcomes and did not assess long-term effects and neurological changes associated with Chiari-like malformation in the rat model. Therefore, longitudinal studies are necessary to better understand the chronic progression of the condition. Fourth, there was a lack of comparative studies between the rat model, other animal models, and human patients. Conducting comparative research is crucial in order to clarify the pathological mechanisms and therapeutic strategies for CLM. Lastly, although we successfully established the rat CLM model, we did not evaluate therapeutic strategies using this model. Therefore, future studies should aim to assess various treatment approaches in order to explore their clinical applicability.

## Conclusions

This study introduces the first experimental animal model of CLM, developed utilizing surgical methods informed by canine data. By employing precise surgical techniques based on canine CCF volume measurements, we successfully generated a rat model that mimics the anatomical and pathological features observed in canine CLM. This model enhances the translational value of our research in the field of veterinary medicine. The reproducible rat CLM model shows promise as a platform for preclinical testing of novel therapeutic interventions, including surgical techniques, pharmacological agents, and rehabilitative strategies specifically designed for veterinary settings. However, its applicability to human Chiari I malformation remains uncertain. Further research is required to explore the generalizability of this model to human conditions and to determine whether it can provide meaningful insights into the pathology and treatment of human Chiari I malformation. Therefore, this model should primarily be regarded as a significant advancement in veterinary research, with potential implications for translational studies only after thorough validation.

## Supporting information

S1 Fig Differences in the rostral and medial fossa (RMF) volume, caudal cranial fossa (CCF) volume, and volume index (VI [CCF/RMF]) between the dog-control (DC) and dog-Chiari (DH) groups. Significant (p value<0.05) differences are marked with *.
(TIF)

S2 Fig Differences in the RMF volume, CCF volume, and VI (CCF/RMF) between the rat-control (RC) and rat-Chiari (RH) groups. Significant (p value<0.05) differences are marked with *. RMF, rostral and medial fossa; CCF, caudal cranial fossa; VI, volume index.
(TIF)

S3 Fig Permutation tests for the caudal cranial fossa (CCF) ratios of the dog-Chiari (DH) group to the dog-control (DC) group and the rat-Chiari (RH) group to the rat-control (RC) group. The permutation test revealed significant differences in the CCF ratio between the DC and DH groups (p = 0.0061). Significant differences were also observed in the CCF ratio between the RC and RH groups (p<0.001). Therefore, it can be concluded that the CCF ratios of dogs and rats exhibit similar characteristics.
(TIF)

S4 Fig Permutation tests for the volume index (VI) ratios of the DH group to the DC group and the RH group to the RC group. The permutation test revealed significant differences in the VI ratio between the DC and DH groups (p = 0.003). Significant differences were also observed in the VI ratio between the RC and RH groups (p<0.001). Therefore, the VI ratios of dogs and rats exhibit similar characteristics. DC, dog-control; DH, dog-Chiari; RH, rat-Chiari; RC, rat-control.
(TIF)

S1 Text Additional information regarding the early stages of research aiming to create experimental animal models of Chiari malformation. (DOCX)

S2 Text Additional information describing Chiari malformations in humans. (DOCX)

S3 Text Additional information describing the surgical details of pinealectomy. (DOCX)
