# Supplementary figures and images for: Surgical modeling of Chiari-like malformation in rats: Insights from canine morphology
Source: PLoS One. 2024 Sep 19;19(9):e0310505. doi: 10.1371/journal.pone.0310505 (PMC11412529; doi:10.1371/journal.pone.0310505)

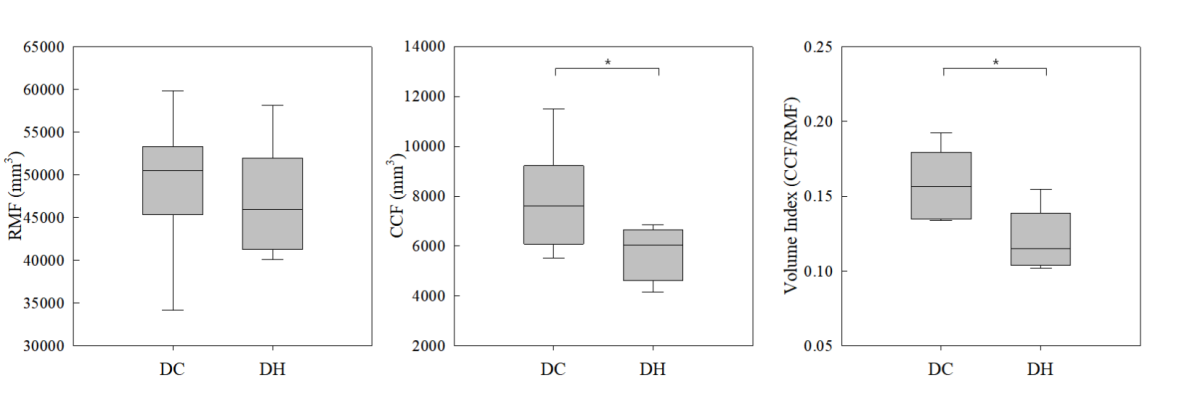

Supplement: S1 Fig — Significant (p value<0.05) differences are marked with *. (TIF) [file pone.0310505.s001.tif]

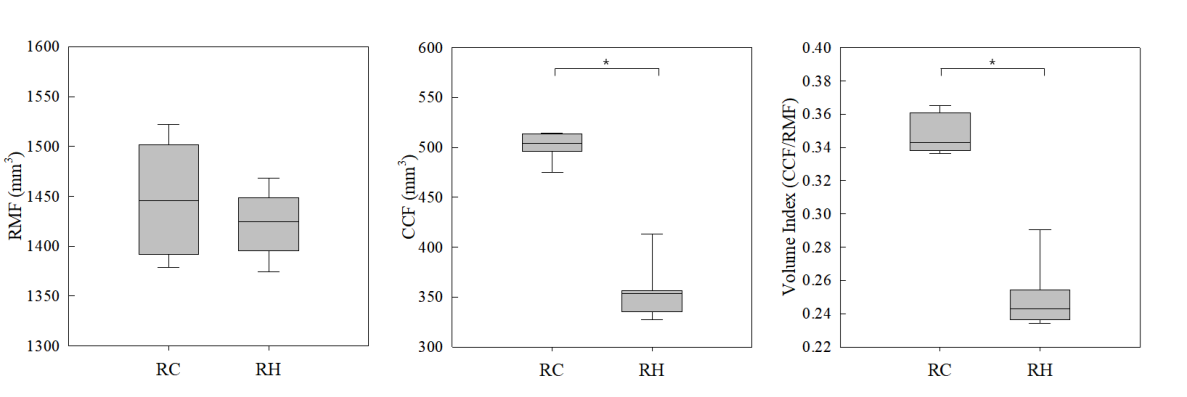

Supplement: S2 Fig — Significant (p value<0.05) differences are marked with *. RMF, rostral and medial fossa; CCF, caudal cranial fossa; VI, volume index. (TIF) [file pone.0310505.s002.tif]

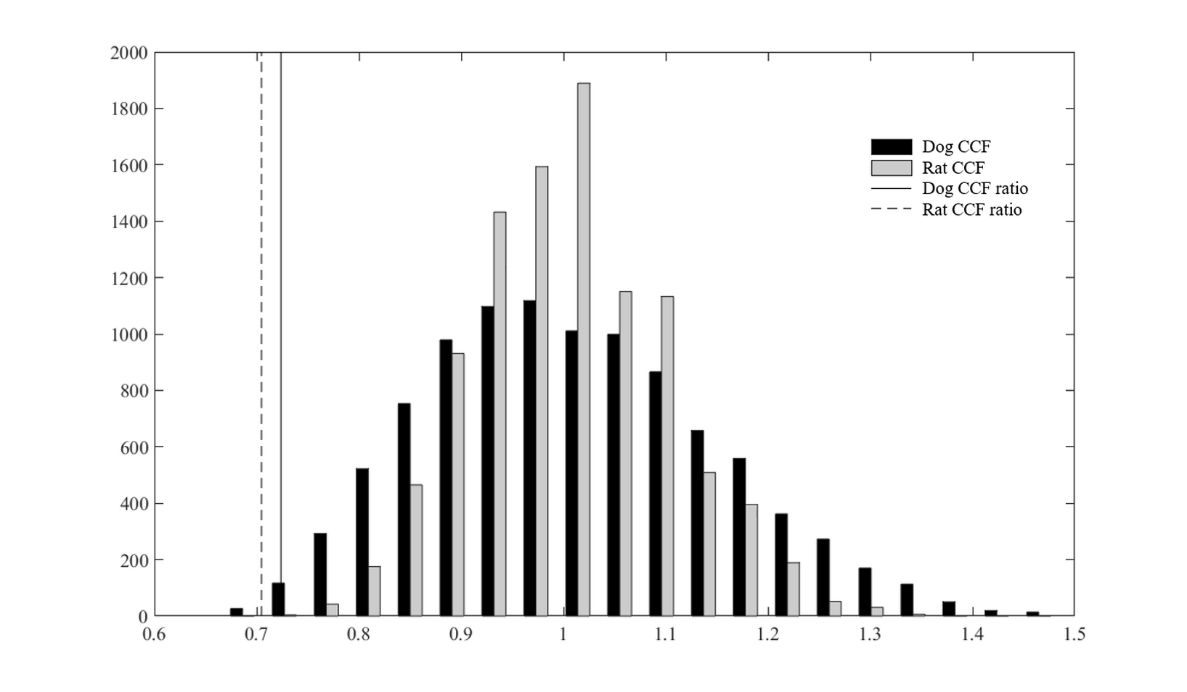

Supplement: S3 Fig — The permutation test revealed significant differences in the CCF ratio between the DC and DH groups (p = 0.0061). Significant differences were also observed in the CCF ratio between the RC and RH groups (p<0.001). Therefore, it can be concluded that the CCF ratios of dogs and rats exhibit similar characteristics. (TIF) [file pone.0310505.s003.tif]

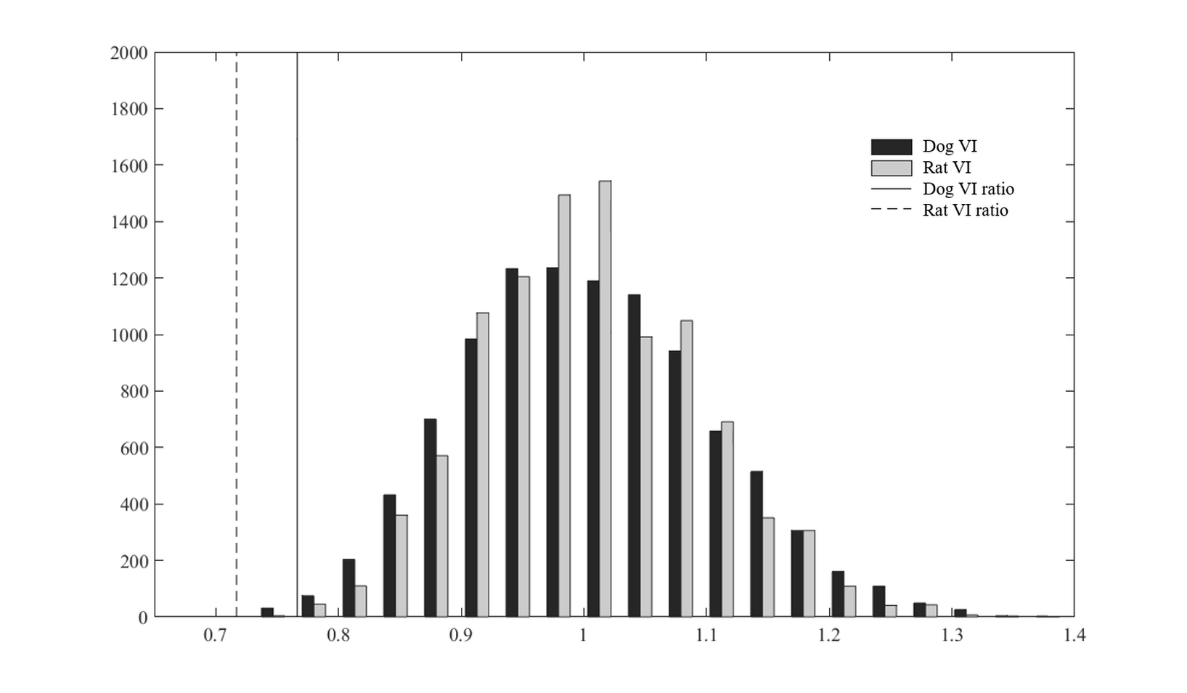

Supplement: S4 Fig — The permutation test revealed significant differences in the VI ratio between the DC and DH groups (p = 0.003). Significant differences were also observed in the VI ratio between the RC and RH groups (p<0.001). Therefore, the VI ratios of dogs and rats exhibit similar characteristics. DC, dog-control; DH, dog-Chiari; RH, rat-Chiari; RC, rat-control. (TIF) [file pone.0310505.s004.tif]
