# Supplementary material for: Surgical modeling of Chiari-like malformation in rats: Insights from canine morphology
Source: PLoS One. 2024 Sep 19;19(9):e0310505. doi: 10.1371/journal.pone.0310505 (PMC11412529; doi:10.1371/journal.pone.0310505)
Supplement: S1 Text — (DOCX) [file pone.0310505.s005.docx]

**S1 Text. Additional information regarding the early stages of research aiming to create experimental animal models of Chiari malformation.**

In 1981, Marin-Padilla and Marin-Padilla proposed that primary paraxial mesoderm insufficiency is the reason for axial skeletal dysraphic disorders, including Chiari malformations, which affect the embryo during or after the closure of neural folds [1]. However, considering that none of the patients exhibited cerebellar herniation into the cervical canal and that the complex nature of this disorder is accompanied by neuraxis anomalies, we believe that our experimental model aligns more closely with Chiari malformation type 2 rather than with type 1. Similar to the work of Marin-Padilla and Marin-Padilla, researchers have continued to develop experimental animal models of Chiari malformations to advance our understanding of these malformations. However, apart from this study conducted by the authors, there has been no successful induction of Chiari type 1 in experimental animals using any other method.
