# Supplementary material for: Surgical modeling of Chiari-like malformation in rats: Insights from canine morphology
Source: PLoS One. 2024 Sep 19;19(9):e0310505. doi: 10.1371/journal.pone.0310505 (PMC11412529; doi:10.1371/journal.pone.0310505)
Supplement: S2 Text — (DOCX) [file pone.0310505.s006.docx]

**S2 Text. Additional information describing Chiari malformations in humans.**

In humans, Chiari malformations are characterized by morphological and anatomical abnormalities in the posterior fossa and hindbrain, including the cerebellum, pons, and medulla oblongata [1]. Chiari types are classified into types 0, 1, 1.5, 2, 3, 4, and 5 based on the severity of morphological and anatomic defects through imaging tests; however, the status of types 0, 1.5, and 5 remains controversial [1]. The widely accepted types include 1, 2, 3, and 4 [1]. Chiari type 1, the most prevalent form, involves the protrusion of the cerebellar tonsil >5 mm below the foramen magnum [1]. Chiari type 2 is a complex disorder with anomalies of the entire neuroaxis characterized by caudal displacement of the cerebellar tonsils, vermis, and brainstem and is often accompanied by spina bifida and myelomeningocele [1,2]. Chiari type 2 exhibits severe alterations in rhombencephalic derivatives along with extensive supratentorial modifications, such as mass intermedia fusion, cortical gyral interdigitation, and “beaking” of the tectum [3]. Chiari types 3 and 4 are rarely encountered in clinical practice, with type 3 involving herniation of the cerebellar medulla into the high cervical or low occipital meningoencephalocele with or without the brainstem and type 4 involving severe cerebellar hypoplasia without herniation of the posterior fossa contents into the foramen magnum, similar to primary cerebellar agenesis [1].
