# Supplementary material for: Surgical modeling of Chiari-like malformation in rats: Insights from canine morphology
Source: PLoS One. 2024 Sep 19;19(9):e0310505. doi: 10.1371/journal.pone.0310505 (PMC11412529; doi:10.1371/journal.pone.0310505)
Supplement: S3 Text — (DOCX) [file pone.0310505.s007.docx]

**S3 Text. Additional information describing the surgical details of pinealectomy.**

Surgically, the anterior border for cutting the occipital bone was established to be 3 mm rostral to the external occipital protuberance and 2 mm caudal to the lambda. It is crucial to determine the location of the lambda since the transverse sinus lies below the dura mater in this region, which increases the risk of death due to heavy bleeding [1]. The occipital bone fragment obtained from the process protrudes by 3 mm and is positioned to overlap the parietal and interparietal bones that make up the skull and is fixed using Vetbond™® (3M Company). At this time, to increase the fixation power, a small hole with a diameter of 0.6 mm was made 1 mm away from the front border of the resected occipital bone fragment using a high-speed dental handpiece equipped with a 0.6 mm diameter carbide bur (FG 1/2, MANI Inc., Japan). This allows Vetbond™® (3M Company) to penetrate effectively. If the protruding part of the occipital bone fragment is less than 3 mm, the fixing power of the Vetbond™® (3M Company) weakens due to reduced contact surface area. As the cerebellum enlarges, the occipital bone fragment loses its fixation, is lifted up, and falls off.
